# Metabolic syndrome and its components predict the biochemical recurrence and adverse pathological features of patients following radical prostatectomy: a propensity score matching study

**DOI:** 10.1186/s12885-023-10507-z

**Published:** 2023-01-14

**Authors:** Zenan Liu, Xuehua Zhu, Jide He, Jian Lu

**Affiliations:** Department of Urology, Peking University Third Hospital, Beijing, China

**Keywords:** Prostate cancer, Radical prostatectomy, Metabolic syndrome, Biochemical recurrence, Adverse pathological features, Propensity score matching

## Abstract

**Background:**

To investigate the predictive value of metabolic syndrome (MetS) and its components in biochemical recurrence (BCR) and adverse pathological features of patients with prostate cancer (PCa) after radical prostatectomy (RP).

**Methods:**

A total of 525 PCa patients who underwent RP between 2010 and 2019 at Peking University Third Hospital were analyzed retrospectively. The Kaplan–Meier method was performed to assess BCR-free survival (BCRFS). Univariate and multivariate Cox regression models and multivariate logistic regression models were conducted to identify the predictive factors of BCRFS and adverse pathological features respectively before and after propensity score matching (PSM).

**Results:**

Enrolled patients were allocated into MetS group (*n* = 136) and non-MetS group (*n* = 389) according to the presence or absence of MetS, and 127 new matched pairs were identified to balance the baseline characteristics after 1:1 PSM. In propensity matched patients, the Kaplan–Meier analysis revealed that MetS (*P* = 0.020), hyperglycemia (*P* = 0.015) and hypertriglyceridemia (*P* = 0.001) were significantly associated with worse BCRFS; the results of multivariate Cox analyses showed that hyperglycemia (*P* = 0.040), hypertriglyceridemia (*P* = 0.017), percentage of positive biopsy cores (*P* = 0.041) and prostate specific antigen (*P* = 0.019) were identified as independent prognostic factors for BCRFS. In addition, hypertriglyceridemia was independently associated with non-organ confined disease (NOCD) (*P* = 0.010), extra-capsular extension (ECE) (*P* = 0.010) and upgrading (*P* = 0.017) in the multivariate logistic analyses.

**Conclusions:**

Hyperglycemia and hypertriglyceridemia are the two effective MetS components both identified as independent risk factors for worse BCRFS after RP, while hypertriglyceridemia was independently associated with NOCD, ECE and upgrading as well.

**Supplementary Information:**

The online version contains supplementary material available at 10.1186/s12885-023-10507-z.

## Introduction

Prostate cancer (PCa) is the most common malignancy cancer of the urinary system and the significant cause of cancer-related deaths in men worldwide. There were an estimated 268,490 new cases of PCa and 34,500 PCa related deaths in 2022 in the United States [1]. In contrast, although the number of newly diagnosed PCa patients in China has been increasing in recent years, the incidence and mortality of PCa are still significantly lower than those in western countries [2, 3]. Apart from genetic factors including ethnic origin and family history, lifestyle‐related factors such as eating habits and sedentariness might also play a significant role in the obvious geographical disparity in PCa risk [4].

Radical prostatectomy (RP) has become the standard treatment for eligible patients due to its superior cancer control and survival benefits [5]. Although most patients are disease-free following RP, nearly 30% of patients continue to experience biochemical recurrence (BCR) during follow-up [6, 7]. Patients with BCR exhibit an extremely worse prognosis due to its association with progression to distant metastases and cancer-specific mortality [8]. Therefore, the assessment of reliable prognostic predictors of BCR after RP is clinically important for guiding clinical decision-making and patient counseling. To date, several traditional clinicopathological factors, such as preoperative prostate‐specific antigen (PSA) levels, Gleason score, tumor stage, surgical margin status, lymph node invasion, extracapsular extension (ECE) and seminal vesicle invasion (SVI) have been identified as prognostic factors for BCR after RP [9]. Although these factors are commonly used to predict BCR‐free survival (BCRFS) after RP, they are unsatisfactory and limited due to their irreversibility. Therefore, more reliable and reversible prognostic factors are required to allow for improvements in disease outcome. In addition, given the significant impact of adverse pathological features on BCR, exploring the potential risk factors of these adverse features can also provide guidance for the preoperative treatment options and effective postoperative management of RP.

Over the past few decades, metabolic syndrome (MetS) has become a prevalent global major health issue, which has attracted extensive attention [10]. MetS are metabolic abnormalities resulting from sedentary lifestyle and excessive diet in a genetically predisposed individual [11], which is characterized by a constellation of metabolic disturbances including abdominal obesity, hypertension, hyperglycemia, hypertriglyceridemia, and low high-density lipoprotein cholesterol (HDL-C) [10]. There is mounting clinical and epidemiologic evidence suggests that MetS is strongly associated with increased risk of PCa incidence [12], cancer progression [13] and poor prognosis [14]. However, regard as the potential association between MetS and BCR or adverse pathological features after RP, the current studies conducted to so far are inconsistent to draw a definitive conclusion. For instance, some studies reported that the presence of MetS was associated with an increased risk of BCR, higher Gleason score, pT3–4 diseases and lymph node involvement after RP [15, 16]. In contrast, another study did not find any significant association between MetS at the time of diagnosis and the risk of BCR or clinicopathological features of PCa after RP [17]. These discrepancies may be explained by the large existence of confounders, the wide heterogeneity of the criteria used for MetS evaluation and ethnicities of the populations.

Therefore, given the inconsistency of existing evidence, the study was aimed to investigate the predictive value of MetS and its components in BCR and adverse pathological features of patients with PCa treated with RP by eliminating potential confounding factors using propensity score matching (PSM), and to provide additional evidence for the current study on the correlation between oncological outcomes after RP and MetS.

## Materials and methods

### Study population

**Fig. 1:**
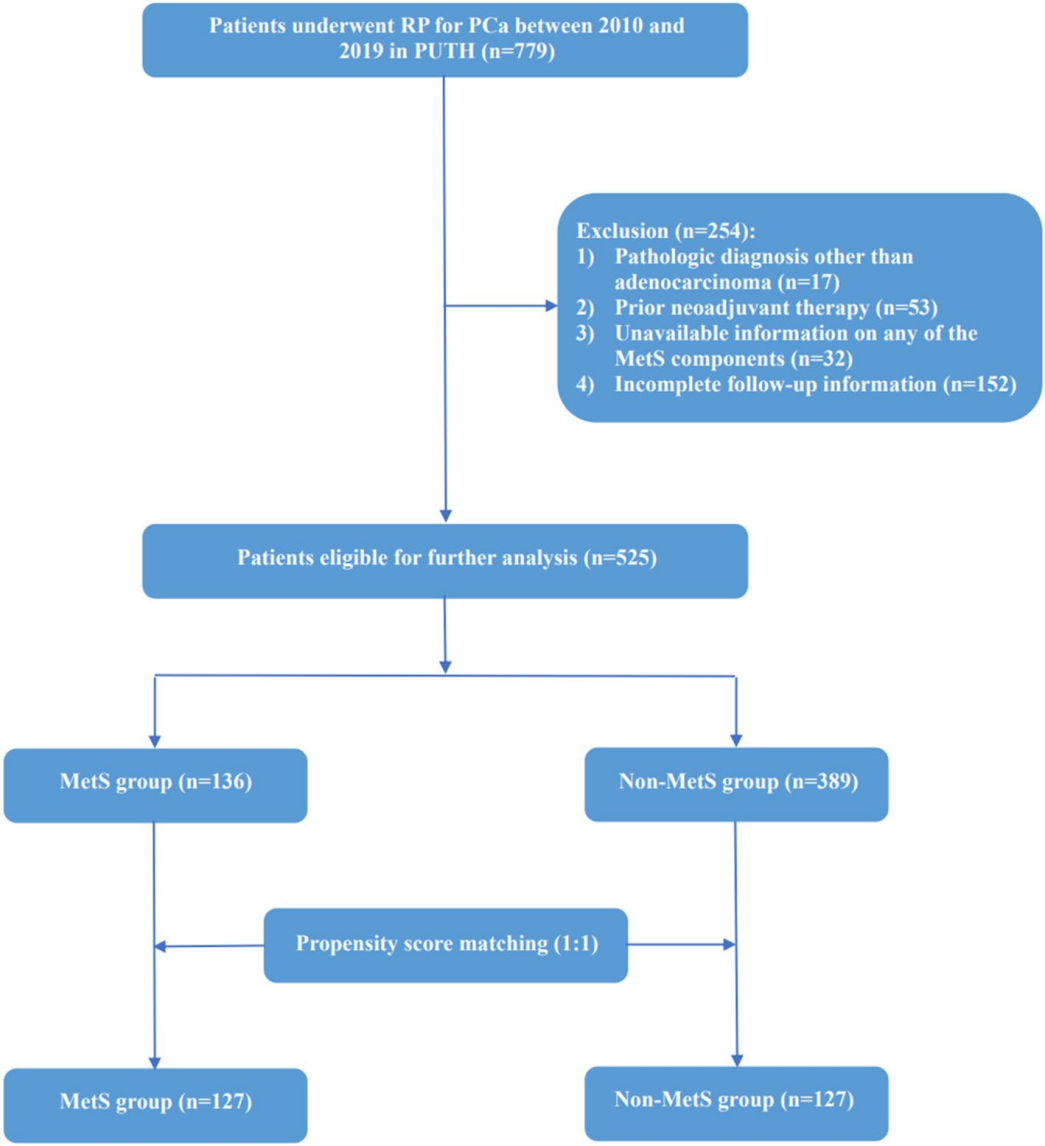
Flow chart of patient selection in the study

The study used the PCa database from the Department of Urology at Peking University Third Hospital (PUTH) with the approval of the Medical Science Research Ethics Committee. A total of 779 consecutive PCa patients who underwent RP between 2010 and 2019 at PUTH were included in the study. For each patient, comprehensive clinicopathologic data and follow-up information were reviewed and collected. Patients were excluded from the study according to the following criteria: histological types other than adenocarcinoma (*n* = 17), prior neoadjuvant therapy (*n* = 53), unavailable information on any of the MetS components (*n* = 32), incomplete follow-up information (*n* = 152). Finally, 525 PCa patients are eligible for further analysis, and the process of patient selection is shown in Fig. 1.

### Data collection and pathological evaluation

The clinical and pathological variables of the enrolled patients were retrospectively collected from the database, including: age, body mass index (BMI), hypertension, hyperglycemia, hypertriglyceridemia, HDL-C, percentage of positive biopsy cores (PPC), preoperative PSA level, pathologic T stage, lymph node status, pathologic Gleason score (GS), surgical margin status, ECE and SVI. The PPC was calculated by dividing the total number of positive biopsy cores by the total number of biopsy cores obtained.

All surgical specimens after RP were processed according to standard pathological procedures. Pathologic report was standardized according to the histological/architectural thresholds proposed by the WHO classification of tumor of the urinary system and male genital organs [18]. The pathologic staging was performed according to the American Joint Committee on Cancer (AJCC) 7^th^ edition TNM staging system [19]. The Gleason scoring system was adopted according to the International Society of Urological Pathology (ISUP) 2005 and 2014 consensus conferences [20]. Non-organ confined disease (NOCD) was defined as pathologic stage ≥ pT3. High grade was defined as pathologic Gleason score ≥ 8 (ISUP ≥ 4). Upgrading was defined as an increase of 1 or more ISUP grade in the Gleason system from biopsy to pathology.

### Metabolic syndrome criteria

Patients were classified as MetS according to the diagnostic criteria from Chinese Medical Association Diabetes Society in 2004 [21] with at least three of the following four components: (1) overweight and/or obesity: BMI ≥ 25 kg/m^2^; (2) hyperglycemia: fasting plasma glucose ≥ 6.1 mmol /L (110 mg/dL) and/or 2-h postprandial plasma glucose ≥ 7.8 mmol/L (140 mg/dL), or drug treatment for diagnosed diabetes mellitus; (3) hypertension: blood pressure ≥ 140/90 mmHg or drug treatment for diagnosed hypertension; (4) dyslipidemia: fasting serum triglyceride (TG) level ≥ 1.7 mmol/L (150 mg/dL) and/or fasting serum HDL-C < 0.9 mmol/L (35 mg/dL) in male and < 1.0 mmol/L (39 mg/dL) in female.

### Follow-up

All patients were followed by serum PSA assessment and clinical visits every 3 months for the first 2 years, semiannually for the next two years, and then annual follow-up thereafter. The primary endpoint of interest in our study was early BCR, defined as present in the event of two consecutive postoperative PSA levels ≥ 0.2 ng/ml [22], and the recurrence date was assigned to the day when the PSA level ≥ 0.2 ng/mL was measured for the first time. BCR-free survival (BCRFS) was calculated from the date of surgery to the date of BCR or the date of last follow-up for those patients who did not experience BCR.

### Statistical analysis

According to the data distribution, categorical variables were expressed as the number of patients with respective percentages, while continuous variables are presented as median and interquartile range (IQR). Between-group comparisons of the MetS patients and non-MetS patients were performed using Student's t test or Mann–Whitney U test for continuous variables and the Pearson’s chi-square test or Fisher’s exact test for categorical variables. We balanced the differences between the patients in the MetS and non-MetS groups by using the method of PSM to obtain matched data. Matching was conducted at a 1:1 fixed ratio using the nearest neighbor method with a caliper value of 0.05 according to the variables of age, hypertension, PPC, preoperative PSA level, pathologic T stage, lymph node status, pathologic Gleason score, surgical margin status, ECE and SVI. BCRFS were estimated using standard Kaplan–Meier methods with Log-rank test. Univariate and multivariate Cox proportional hazards regression models were performed to evaluate the associations of MetS and individual components with BCRFS, and the results were presented as hazards ratio (HR) and 95% confidence interval (95% CI). The associations of MetS and its individual components with adverse pathological features (NOCD [≥ pT3], lymph node invasion, high-grade [ISUP ≥ 4], upgrading, positive surgical margin, ECE and SVI) after RP by using multivariate binary logistic regression model, and the results were summarized as odds ratio (OR) with respective 95% CI. All statistical analysis were performed using IBM SPSS Statistics 26.0. Two-sided *P* values < 0.05 were considered statistically significant.

## Results

### Patient characteristics

According to the inclusion and exclusion criteria, 525 patients treated with RP were included and they were allocated into MetS group (*n* = 136) and non-MetS group (n = 389) based on the presence or absence of MetS. The overall prevalence of obesity, hypertension, hyperglycemia, hypertriglyceridemia and low HDL-C in the MetS components were 46.5%, 48.6%, 29.0%, 28.2% and 17.5%, respectively. A matched analysis was performed according to propensity scores at a 1:1 fixed ratio to adjust for heterogeneity in the MetS group and non-MetS group, and finally we obtained 127 new matched pairs. There is a well-matched distribution with respect to clinicopathologic characteristics in the adjusted analysis after case matching between patients in the MetS and non-MetS groups. The clinicopathologic characteristics of the patients before and after PSM are shown in Table 1.

**Table 1:** Clinicopathological characteristics of PCa patients treated with RP before and after PSM

Characteristics	All patients (*n* = 525)			Propensity matched patients (*n* = 254)		
	**MetS (*n* = 136)**	**Non-MetS (*n* = 389)**	***P* value**	**MetS (*n* = 127)**	**Non-MetS (*n* = 127)**	***P* value**
Age (years), median (IQR)	68 (64–74)	70 (65–75)	0.083	69 (65–75)	71 (67–75)	0.099
BMI (kg/m^2^), n (%)			< 0.001			< 0.001
< 25	23 (16.9%)	258 (66.3%)		23 (18.1%)	82 (64.6%)	
≥ 25	113 (83.1%)	131 (33.7%)		104 (81.9%)	45 (35.4%)	
Hypertension, n (%)			< 0.001			1.000
No	18 (13.2%)	252 (64.8%)		18 (14.2%)	18 (14.2%)	
Yes	118 (86.8%)	137 (35.2%)		109 (85.8%)	109 (85.8%)	
Hyperglycemia, n (%)			< 0.001			< 0.001
No	48 (35.3%)	325 (83.5%)		41 (32.3%)	106 (83.5%)	
Yes	88 (64.7%)	64 (16.5%)		86 (67.7%)	21 (16.5%)	
Hypertriglyceridemia, n (%)			< 0.001			< 0.001
No	47 (34.6%)	330 (84.8%)		45 (35.4%)	109 (85.8%)	
Yes	89 (65.4%)	59 (15.2%)		82 (64.6%)	18 (14.2%)	
Low HDL-C, n (%)			< 0.001			< 0.001
No	88 (64.7%)	345 (88.7%)		80 (63.0%)	118 (92.9%)	
Yes	48 (35.3%)	44 (11.3%)		47 (37.0%)	9 (7.1%)	
PPC (%), median (IQR)	41.7 (23.1–66.7)	41.7 (24.1–61.5)	0.447	41.7 (23.1–66.7)	41.7 (23.1–61.5)	0.601
Preoperative PSA (ng/mL), n (%)			0.294			0.677
< 20	94 (69.1%)	287 (73.8%)		89 (70.1%)	92 (72.4%)	
≥ 20	42 (30.9%)	102 (26.2%)		38 (29.9%)	35 (27.6%)	
Pathologic T Stage, n (%)			0.070			0.575
≤ T2c	69 (50.7%)	224 (57.6%)		68 (53.5%)	76 (59.8%)	
T3a	47 (34.6%)	95 (24.4%)		39 (30.7%)	35 (27.6%)	
≥ T3b	20 (14.7%)	70 (18.0%)		20 (15.7%)	16 (12.6%)	
Lymph node Status, n (%)			0.979			0.848
N0/Nx	117 (86.0%)	335 (86.1%)		111 (87.4%)	112 (88.2%)	
N +	19 (14.0%)	54 (13.9%)		16 (12.6%)	15 (11.8%)	
Pathologic Gleason score, n (%)			0.692			0.296
≤ 3 + 4	45 (33.1%)	136 (35.0%)		42 (33.1%)	50 (39.4%)	
≥ 4 + 3	91 (66.9%)	253 (65.0%)		85 (66.9%)	77 (60.6%)	
Surgical margin, n (%)			0.414			0.609
Negative	81 (59.6%)	247 (63.5%)		74 (58.3%)	78 (61.4%)	
Positive	55 (40.4%)	142 (36.5%)		53 (41.7%)	49 (38.6%)	
ECE, n (%)			0.180			0.310
Absent	70 (51.5%)	226 (58.1%)		69 (54.3%)	77 (60.6%)	
Present	66 (48.5%)	163 (41.9%)		58 (45.7%)	50 (39.4%)	
SVI, n (%)			0.307			0.338
Absent	118 (86.8%)	323 (83.0%)		109 (85.8%)	114 (89.8%)	
Present	18 (13.2%)	66 (17.0%)		18 (14.2%)	13 (10.2%)	

*BMI* body mass index, *ECE* extra-capsular extension, *HDL-C* high density lipoprotein cholesterol, *IQR* interquartile range, *MetS* metabolic syndrome, *PCa* prostate cancer, *PPC* percentage of positive biopsy cores, *PSA* prostate specific antigen, *PSM* propensity score matching, *RP* radical prostatectomy, *SVI* seminal vesicle invasion

### Survival analysis before PSM

In all patients, the median follow-up period was 36.6 months (IQR: 18.2–60.8 months), BCR was experienced in 45 (33.1%) patients with MetS and 95 (24.4%) patients without MetS. The median BCRFS time was 26.6 months, the 3-year and 5-year BCRFS probabilities of the patient with MetS were 62.4% and 56.2% respectively, while those of patients without MetS were 73.3% and 66.2%, respectively.

**Fig. 2:**
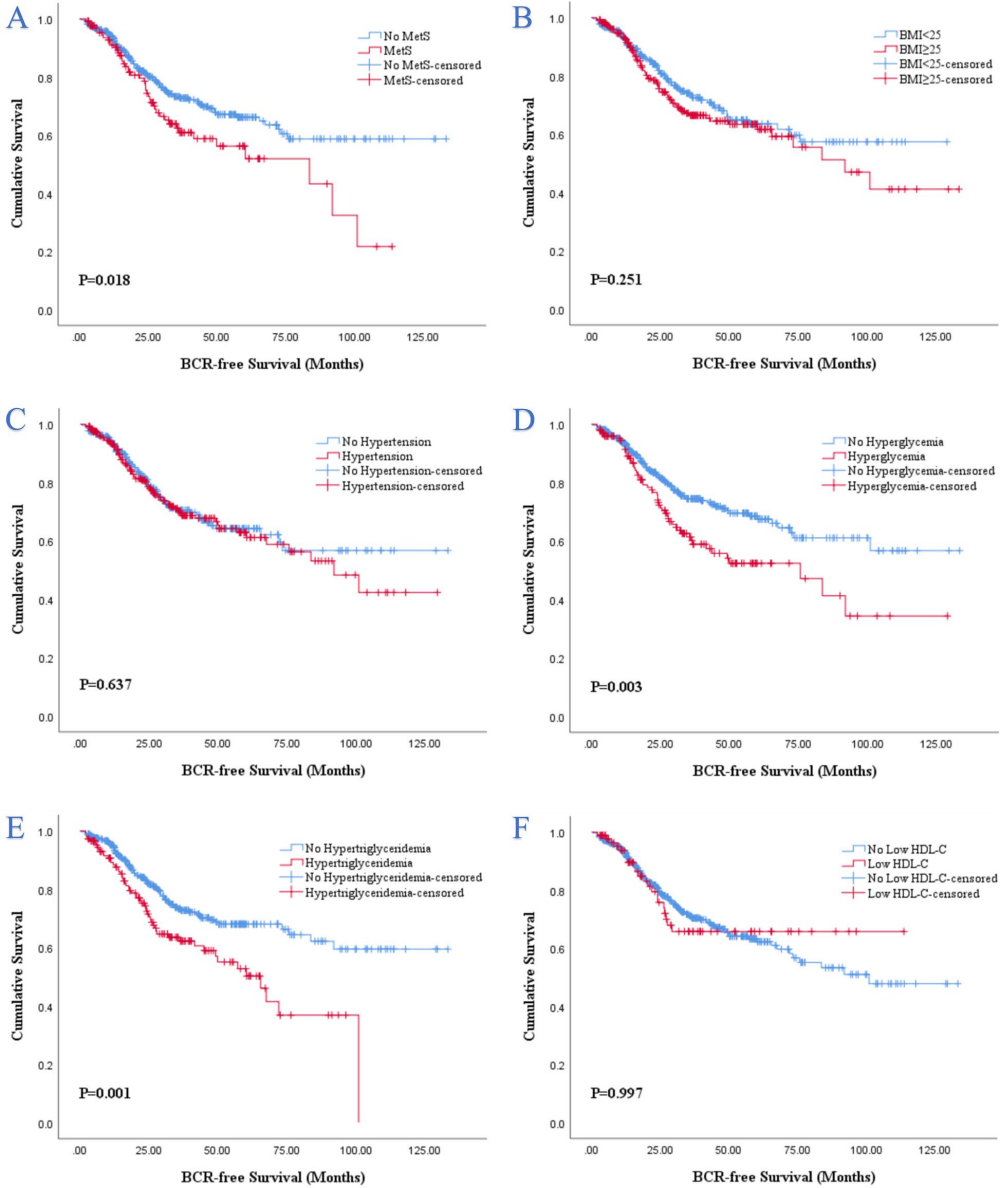
Kaplan–Meier survival analysis of BCR-free survival stratified by MetS and its components before PSM. **A** MetS and non-MetS; **B** BMI < 25 and BMI ≥ 25; **C** hypertension and no hypertension; **D** hyperglycemia and no hyperglycemia; **E** hypertriglyceridemia and no hypertriglyceridemia; **F** low HDL-C and no low HDL-C. BCR, biochemical recurrence; BMI, body mass index; HDL-C, high density lipoprotein cholesterol; MetS, metabolic syndrome; PSM, propensity score matching

There was statistical significance can be observed in BCRFS for MetS (Fig. 2A), hyperglycemia (Fig. 2D) and hypertriglyceridemia (Fig. 2E) in the Kaplan–Meier analysis. The presence of MetS (*P* = 0.018), hyperglycemia (*P* = 0.003) and hypertriglyceridemia (*P* = 0.001) were significantly associated with worse BCRFS compared with the absence of MetS, hyperglycemia and hypertriglyceridemia, respectively. Unfortunately, other individual MetS components in addition to above did not show a significant association with BCRFS (Fig. 2B-C, F).

The results of univariate Cox analyses demonstrated that MetS, hyperglycemia, hypertriglyceridemia, PPC, preoperative PSA level, pathologic T stage, pathologic GS, surgical margin status, ECE and SVI were significantly associated with BCRFS (*P* < 0.05; Table 2). Incorporated the above factors into multivariate Cox regression analysis, hyperglycemia (*P* = 0.014), hypertriglyceridemia (*P* = 0.011) and PPC (*P* = 0.027) were identified as independent prognostic factors for BCRFS (Table 2).

**Table 2:** Univariate and multivariate analysis of prognostic factors using the Cox proportional hazards model for BCRFS in RP patients

Variables	Before PSM				After PSM			
	**Univariate analysis**		**Multivariate analysis**		**Univariate analysis**		**Multivariate analysis**	
	**HR (95%CI)**	***P* value**	**HR (95%CI)**	***P* value**	**HR (95%CI)**	***P* value**	**HR (95%CI)**	***P* value**
Age (years)	0.986 (0.964–1.008)	0.208			0.980 (0.946–1.015)	0.260		
MetS								
No	Ref		Ref		Ref		Ref	
Yes	1.529 (1.071–2.182)	0.019	0.913 (0.579–1.439)	0.694	1.753 (1.085–2.833)	0.022	0.814 (0.414–1.600)	0.550
BMI (kg/m^2^)								
< 25	Ref				Ref			
≥ 25	1.214 (0.871–1.691)	0.252			1.356 (0.837–2.195)	0.216		
Hypertension								
No	Ref				Ref			
Yes	1.083 (0.778–1.509)	0.637			1.264 (0.626–2.552)	0.513		
Hyperglycemia								
No	Ref		Ref		Ref		Ref	
Yes	1.676 (1.193–2.354)	0.003	1.613 (1.100–2.366)	0.014	1.774 (1.110–2.835)	0.017	1.792 (1.027–3.125)	0.040
Hypertriglyceridemia								
No	Ref		Ref		Ref		Ref	
Yes	1.783 (1.268–2.506)	0.001	1.669 (1.126–2.474)	0.011	2.193 (1.366–3.519)	0.001	2.076 (1.138–3.787)	0.017
Low HDL-C								
No	Ref				Ref			
Yes	0.999 (0.628–1.590)	0.997			0.801 (0.420–1.527)	0.500		
PPC (%)	1.015 (1.009–1.021)	< 0.001	1.008 (1.001–1.015)	0.027	1.017 (1.009–1.026)	< 0.001	1.010 (1.000–1.020)	0.041
Preoperative PSA (ng/mL)								
< 20	Ref		Ref		Ref		Ref	
≥ 20	2.048 (1.457–2.877)	< 0.001	1.431 (0.976–2.098)	0.067	2.753 (1.711–4.430)	< 0.001	1.931 (1.115–3.344)	0.019
Pathologic T Stage								
≤ T2c	Ref		Ref		Ref		Ref	
T3a	1.288 (0.869–1.908)	0.208	5.206 (0.579–46.837)	0.141	1.398 (0.819–2.385)	0.220	1.372 (0.077–24.440)	0.830
≥ T3b	2.060 (1.359–3.123)	0.001	3.852 (0.900–16.484)	0.069	2.389 (1.277–4.470)	0.006	2.684 (0.356–20.248)	0.338
Lymph node Status								
N0/Nx	Ref				Ref			
N +	1.073 (0.680–1.694)	0.762			1.070 (0.561–2.043)	0.837		
Pathologic Gleason score								
≤ 3 + 4	Ref		Ref		Ref		Ref	
≥ 4 + 3	2.139 (1.433–3.192)	< 0.001	1.527 (0.974–2.394)	0.065	2.060 (1.195–3.550)	0.009	1.249 (0.650–2.400)	0.504
Surgical margin								
Negative	Ref		Ref		Ref			
Positive	1.543 (1.107–2.152)	0.011	1.133 (0.790–1.624)	0.498	1.460 (0.916–2.327)	0.112		
ECE								
Absent	Ref		Ref		Ref		Ref	
Present	1.491 (1.069–2.079)	0.019	0.167 (0.019–1.476)	0.107	1.624 (1.017–2.592)	0.042	0.745 (0.045–12.281)	0.837
SVI								
Absent	Ref		Ref		Ref		Ref	
Present	1.887 (1.270–2.804)	0.002	1.815 (0.298–11.057)	0.518	2.190 (1.171–4.094)	0.014	0.611 (0.074–5.072)	0.648

*BCRFS* BCR-free survival, *BMI* body mass index, *CI* confidence interval, *ECE* extra-capsular extension, *HDL-C* high density lipoprotein cholesterol, *HR* hazard ratio, *MetS* metabolic syndrome, *PPC* percentage of positive biopsy cores, *PSA* prostate specific antigen, *PSM* propensity score matching, *Ref* reference, *RP* radical prostatectomy, *SVI* seminal vesicle invasion

### Survival analysis after PSM

In propensity matched patients, the median follow-up period was 37.1 months (IQR: 18.2–64.9 months), BCR was experienced in 41 (32.3%) patients with MetS and 30 (23.6%) patients without MetS. The median BCRFS time was 26.4 months, the 3-year and 5-year BCRFS probabilities of the patient with MetS were 63.3% and 56.1% respectively, while those of patients without MetS were 75.0% and 68.4%, respectively.

**Fig. 3:**
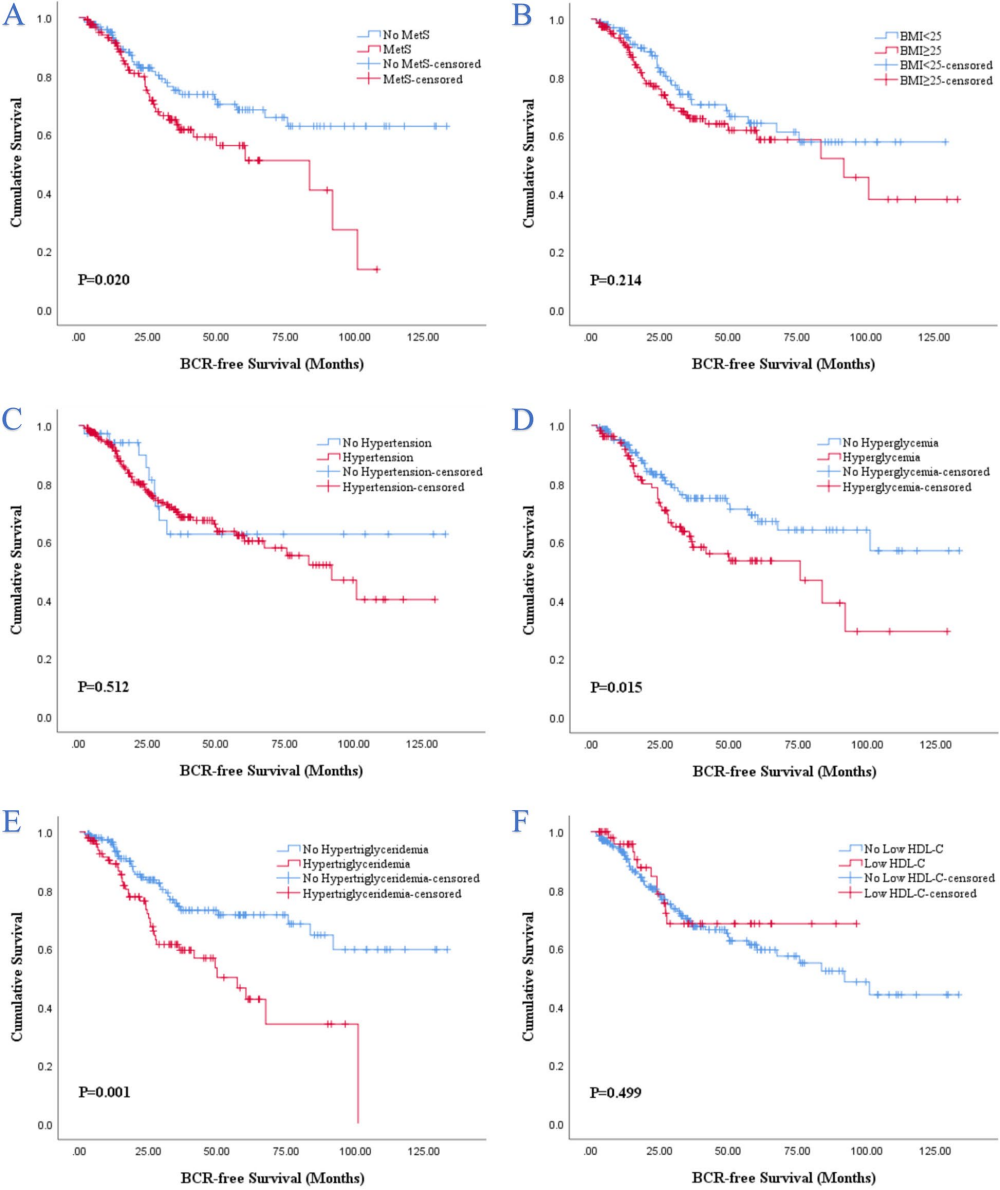
Kaplan–Meier survival analysis of BCR-free survival stratified by MetS and its components after PSM. **A** MetS and non-MetS; **B** BMI < 25 and BMI ≥ 25; **C** hypertension and no hypertension; **D** hyperglycemia and no hyperglycemia; **E** hypertriglyceridemia and no hypertriglyceridemia; **F** low HDL-C and no low HDL-C. BCR, biochemical recurrence; BMI, body mass index; HDL-C, high density lipoprotein cholesterol; MetS, metabolic syndrome; PSM, propensity score matching

We also investigated the effects of MetS and its components on the BCRFS in the propensity matched patients, and the results of Kaplan–Meier analysis and Cox regression analyses were similar to those before PSM. The Kaplan–Meier analysis demonstrated that the presence of MetS (*P* = 0.020; Fig. 3A), hyperglycemia (*P* = 0.015; Fig. 3D) and hypertriglyceridemia (*P* = 0.001; Fig. 3E) were still significantly associated with worse BCRFS compared with the absence of MetS, hyperglycemia and hypertriglyceridemia respectively, despite of no significant association with BCRFS could be observed in other individual MetS components (Fig. 3B-C, F). The results of univariate Cox analyses also revealed that MetS, hyperglycemia, hypertriglyceridemia, PPC, preoperative PSA level, pathologic T stage, pathologic GS, ECE and SVI were significantly associated with BCRFS (*P* < 0.05; Table 2). Incorporated the above factors into multivariate Cox regression analysis, hyperglycemia (*P* = 0.040), hypertriglyceridemia (*P* = 0.017), PPC (*P* = 0.041) and PSA (*P* = 0.019) were identified as independent prognostic factors for BCRFS (Table 2).

### Subgroup analysis for BCRFS in propensity matched patients

We further performed subgroup analysis to explore the effect of MetS and its components on BCRFS using multivariate Cox analyses in propensity matched patients according to the following variables: 1) age (< 70 vs ≥ 70); 2) BMI (< 25 vs ≥ 25); 3) preoperative PSA (< 20 vs ≥ 20); 4) pathologic T Stage (OCD vs NOCD); 5) pathologic Gleason score [low (GS < 8) vs high (GS ≥ 8)]; 6) surgical margin (negative vs positive). The results revealed that hyperglycemia and/or hypertriglyceridemia were still identified as independent prognostic factors for BCRFS in the most subgroups, especially in the BMI ≥ 25, organ confined disease (OCD) and low GS subgroups (Supplementary Table 1). In addition, BMI was found to be significantly associated with worse BCRFS in the age ≥ 70 (*P* = 0.028) and NOCD (*P* = 0.047) subgroups (Supplementary Table 1).

### The effect of MetS and its components on adverse pathological features in propensity matched patients

Multivariate logistic regression analyses were performed to explore the potential association between MetS and its components and adverse pathological features in propensity matched patients. From the multivariate regression tests, only hypertriglyceridemia was identified as independent prognostic factor for NOCD (*P* = 0.010), ECE (*P* = 0.010) and upgrading (*P* = 0.017) in the components of MetS (Table 3). Hypertriglyceridemia was independently associated with reduced risk of NOCD (OR = 0.376, 95% CI = 0.179–0.792) and ECE (OR = 0.374; 95% CI = 0.177–0.788), while it was independently associated with increased risk of upgrading (OR = 2.433, 95% CI = 1.175–5.036). The reduced association of hypertriglyceridemia with NOCD and ECE appear to a survival benefit, while the increased risk of upgrading points to a worse prognosis.

**Table 3:** Multivariate analysis of MetS and its components for adverse pathological features using the logistic regression models in RP patients after PSM

Variables	Multivariate analysis	
	**OR (95%CI)**	***P* value**
NOCD		
MetS	2.505 (0.880–7.130)	0.085
BMI	0.929 (0.452–1.911)	0.842
Hypertension	0.854 (0.363–2.008)	0.718
Hyperglycemia	0.667 (0.312–1.428)	0.297
Hypertriglyceridemia	0.376 (0.179–0.792)	0.010
Low HDL-C	1.191 (0.572–2.482)	0.640
Lymph node invasion		
MetS	2.144 (0.485–9.484)	0.315
BMI	0.808 (0.294–2.222)	0.680
Hypertension	0.612 (0.193–1.936)	0.403
Hyperglycemia	0.774 (0.270–2.219)	0.634
Hypertriglyceridemia	0.471 (0.161–1.378)	0.169
Low HDL-C	0.977 (0.344–2.780)	0.966
High grade		
MetS	1.082 (0.392–2.992)	0.879
BMI	1.655 (0.815–3.360)	0.163
Hypertension	0.809 (0.353–1.855)	0.617
Hyperglycemia	1.306 (0.624–2.730)	0.479
Hypertriglyceridemia	0.558 (0.269–1.157)	0.117
Low HDL-C	0.634 (0.303–1.325)	0.225
Upgrading		
MetS	0.565 (0.204–1.567)	0.273
BMI	1.277 (0.629–2.590)	0.499
Hypertension	0.672 (0.303–1.490)	0.328
Hyperglycemia	1.014 (0.483–2.128)	0.971
Hypertriglyceridemia	2.433 (1.175–5.036)	0.017
Low HDL-C	0.869 (0.420–1.796)	0.704
Positive surgical margin		
MetS	0.729 (0.271–1.963)	0.531
BMI	1.385 (0.691–2.776)	0.358
Hypertension	1.080 (0.478–2.440)	0.853
Hyperglycemia	1.140 (0.553–2.350)	0.723
Hypertriglyceridemia	1.685 (0.837–3.396)	0.144
Low HDL-C	0.985 (0.485–2.001)	0.968
ECE		
MetS	2.394 (0.841–6.810)	0.102
BMI	0.972 (0.472–1.999)	0.938
Hypertension	0.834 (0.355–1.960)	0.677
Hyperglycemia	0.734 (0.344–1.567)	0.424
Hypertriglyceridemia	0.374 (0.177–0.788)	0.010
Low HDL-C	1.126 (0.540–2.349)	0.751
SVI		
MetS	3.630 (0.643–20.504)	0.145
BMI	0.405 (0.125–1.310)	0.131
Hypertension	0.684 (0.179–2.609)	0.579
Hyperglycemia	0.870 (0.280–2.701)	0.810
Hypertriglyceridemia	0.472 (0.136–1.634)	0.236
Low HDL-C	0.645 (0.206–2.019)	0.451

*BMI* body mass index, *CI* confidence interval, *ECE* extra-capsular extension, *HDL-C* high density lipoprotein cholesterol, *MetS* metabolic syndrome, *NOCD* non-organ confined disease, *OR* odds ratio, *PSM* propensity score matching, *RP* radical prostatectomy, *SVI* seminal vesicle invasion

## Discussion

To the best of our knowledge, this is the first study to comprehensively explore whether MetS or its components influence BCR as well as adverse pathological features of patients following RP by using PSM. Balancing the differences of baseline characteristics between the patients in the MetS and non-MetS groups and eliminating potential confounding factors by using the method of PSM is an advantage of our study. We found that the presence of MetS were significantly associated with worse BCRFS in Kaplan–Meier analysis, while hyperglycemia and hypertriglyceridemia were identified as independent prognostic factors for BCRFS by multivariate Cox analysis adjusted for other clinicopathological factors. BMI was also found to be significantly associated with worse BCRFS in population with age ≥ 70 and with NOCD in the subgroup analysis. In addition, we performed further analyses for exploring the association between MetS and its components and adverse pathological features, the results revealed that hypertriglyceridemia was the only component independently associated with NOCD (≥ pT3a), ECE and upgrading in propensity matched patients.

We observed that the prevalence of MetS in RP population is 25.9% in our study, which is consistent with previous studies that reported incidence rate ranging from 18%-30% [23–25]. The risk of morbidity and mortality associated with PCa varies in different countries, with the highest in Western countries and the lowest in Asian countries [26]. The epidemiological characteristics of MetS are also similar to those of PCa that are manifested by an obviously higher incidence rate in Western countries compared with Asian countries [27], which indicates that MetS plays a significant role in pathogenesis of PCa. In addition, the presence of MetS was supposed to be closely associated with increased incidence, aggressiveness and unfavorable prognosis of PCa as well, while several potential molecular mechanisms and metabolic pathways are well characterized including insulin resistance and comorbid hyperinsulinemia [dysregulation of the insulin-like growth factor (IGF) signaling pathway] [28], pro-inflammatory condition and abnormal adipokines levels [16, 29], and a microenvironment conducive to tumor formation induced by adipose tissue [30, 31]. However, the current knowledge might represent a small part of the biological mechanisms underlying these associations, and further studies are still warranted.

In addition to traditional clinicopathological factors such as preoperative PSA level, tumor stage, GS and positive surgical margin, the results of our study also showed that MetS was significantly associated with worse BCRFS after RP in PCa patients, although the predictive role was not independent. It indicated that there is a higher incidence of BCR after RP among patients with MetS. However, current researches on the association between MetS and BCR after RP remain controversial. Shiota M et al. [15] considered that the feature of MetS is an independent risk factor for BCR after RP among Japanese men. Similarly, Castillejos-Molina R et al. [25] also concluded that MetS is independently associated with the risk of biochemical progression in both OCD and locally advanced PCa. In contrast, neither the result of Xu X et al. [32] and Morlacco A et al. [33] showed any relationship between the presence of MetS and BCR among the cohort in China and Europe, respectively. These discrepancies might result from the wide heterogeneity of MetS definition and ethnicities of the populations. In addition, given the significant impact of adverse pathological features on BCR, quantities of studies have investigated and revealed the close association between MetS and its components and adverse pathological features after RP, despite of the considerable differences between the findings of various studies. A retrospective study consisted of 1016 Chinese patients with PCa who received RP revealed that MetS indicated an increased risk of prostatectomy GS ≥ 8, pT3-4 disease and lymph node involvement [16]. On the contrary, this positive relationship was not found in studies by Beebe-Dimmer JL et al. [34] and Xu X et al. [32]. Furthermore, MetS also represents a significant risk factor for positive surgical margin [23], upgrading and upstaging after RP [35]. However, there was no statistical significance between MetS and any postoperative adverse pathological features after adjusting other clinicopathological variables using multivariate analysis in our study. It could be explained by the fact that MetS is a syndrome consisting of at least three components, and the individual component may exert antagonistic roles (like positive and negative functions cancel each other out), thus ultimately manifesting as a meaningless composite outcome.

Apart from MetS itself, meaningful outcomes were found when focus on individual MetS components alone in further analysis as well. hyperglycemia and hypertriglyceridemia were the only two MetS components both identified as independent risk factors for increased risk of BCR after RP. Our findings on hyperglycemia can be supported by previous other studies with similar conclusions. The result of a case–control study showed that diabetes was significantly associated with an increased likelihood of BCR after RP regardless of metformin use [36]. Wright JL et al. [37] also concluded that glucose levels at the time of PCa diagnosis is an independent predictor of BCR for men undergoing RP for localized disease. This positive association comes in contrast to other researches. Rieken M et al. [38] could not detect a significant association between diabetes mellitus and increased risk of postoperative BCR in a cohort of 6,863 RP patients. More recent studies also failed to establish a significant link between hyperglycemia and BCR [39, 40]. These contradictory findings might be related to the fact that diabetes could interact with PCa cells at different levels. On the one hand, diabetes may have a potential protective effect against the progression of PCa by reducing the activity of IGF‐I and testosterone levels [41, 42]. On the other hand, elevated glucose levels accompanied by hyperinsulinemia represent the underlying mechanism by which the PCa development and BCR could be induced by diabetes [43]. Therefore, further well-controlled clinical researches with large sample sizes are still warranted to provide more evidence regarding the association between hyperglycemia and BCR with corresponding numerous mechanisms.

As for hypertriglyceridemia, the existing data is both scarce and divergent. A meta-analysis integrating 12 articles involving 11,108 patients concluded that there was no significant correlation between hypertriglyceridemia with BCR after RP [44]. Inconsistent with the above results, our study demonstrated that patients with hypertriglyceridemia was significantly associated with worse BCRFS. This was also further confirmed by Kaplan–Meier analysis, which revealed an increased risk of BCR in patients with hypertriglyceridemia. In addition, further analysis also showed that hypertriglyceridemia is independently association high risk of upgrading after RP. The results of Arthur R et al. [45] and Hayashi et al. [46] about that hypertriglyceridemia was positively associated with high-grade PCa (GS ≥ 8), which contribute to support our conclusions indirectly. The underlying mechanisms for this positive risk correlation could be explained by the results of several experimental studies using in vitro models [47]. However, several interesting results in our study can also be observed that hypertriglyceridemia was the protective factor significantly associated reduced risk of NOCD (≥ pT3) and ECE, which is inconsistent with Zheng X et al. [44] who suggested that hypertriglyceridemia was linked with higher risk of pT ≥ T3. Kang M et al. [48] also revealed that preoperative hypertriglyceridemia was significantly associated with a reduced risk of BCR after RP. Serum cholesterol levels can be reduced by cancer cells, which provides a mechanism basis for this positive protective association. Malignant cells grow and proliferate rapidly, which required consuming large amounts of serum cholesterol for the biosynthesis of new cell membrane to meet accelerated metabolic turnover [49]. In this respect, patients with unfavorable tumor phenotype are considered likely to have lower baseline cholesterol due to abundant storage of lipids in their cancer cells [48]. Thus, further studies are required to verify whether there is a significant accumulation of intracellular triglyceride in the surgical specimens of PCa patients with BCR to provide more convincing evidence for our conclusion.

At present, it has been proposed that higher BMI increases the risk of tumor aggressiveness, BCR and cancer-specific mortality of PCa in several studies [50–52]. In contrast, the primary analysis of our study did not show any association between overweight and/or obesity (BMI ≥ 25) and BCR as well as adverse pathological features in RP patients, which is consistent with most previous studies [53–55]. The differences may be related to variations in the cutoff values of BMI and treatment selection. Nevertheless, we observed the independent predictive role of BMI in the patients with age ≥ 70 and with NOCD in subgroup analysis, which suggests that BMI might influence the outcomes in a distinct manner from dyslipidemia. On the one hand, obese men more often have lower testosterone levels, low testosterone concentrations are thought to promote the development of aggressive forms of PCa through a carcinogenic inflammatory pathway [52]. As testosterone levels in elderly men decline with age gradually, this unfavorable mechanism of obesity will be further exacerbated. On the other hand, due to technical difficulties of performing surgery, obesity is thought to be associated with a higher risk of positive surgical margins after RP, thereby increasing the likelihood of BCR [56, 57]. When patients accompanied by NOCD, the presence of ECE and/or SVI would inevitably increase the possibility of positive surgical margin and further in the risk of BCR in collaboration with obesity.

Our findings have several clinical implications. First off, our results provide evidence to support the role of MetS and its components (especially hyperglycemia and hypertriglyceridemia) in preoperative risk stratification for BCR and adverse pathological features after RP by using the method of PSM. The findings may provide a research direction about new strategies or approaches for PCa treatment, such as diet, exercise and medications (like statins and metformin) for MetS, which contribute to improve the prognosis of PCa by reversing MetS. Then, the natural history of BCR after RP could be long but variable. The determination of risk assessment factors such as preoperative MetS, hyperglycemia, hypertriglyceridemia and BMI could be conducive to recognize patients with high-risk BCR after RP, and may benefit from aggressive salvage treatment. In addition, accurate preoperative staging is vital for the management strategy of PCa. Thus, urgent improvement of the current situation of a great quantity of tumors are over- or under-staged is needed [58]. Although no association between MetS and adverse pathological features can be found in this study, this information of the significant relationship between hypertriglyceridemia and the features of NOCD and ECE would contribute to predict stage in PCa patients accurately.

There are several limitations of the study that need to be acknowledged. First and foremost, the retrospective design is the primary limitation, which only allowed us to evaluate the temporal link between MetS and BCR, thereby causal inferences are limited, and the potential selection bias can not be ignored due to the retrospective nature as well. Second, this study mainly focused on the Chinese population and we used the diagnostic criteria of MetS that are most suitable for the Chinese population, which might affect the applicability of research results in other ethnic populations. Meanwhile, we adopted BMI rather than waist circumferences to define overweight or obesity while waist circumference may be more closely related to metabolic changes compared with BMI [59], which could lead to misclassification. Last but not least, additional potential confounding factors, such as family history of cancer, dietary habits, physical activities and drug treatment including stains, aspirin or metformin were not evaluated in the study. Data on these factors would be of great important since they are known to play a vital role in the association between MetS and BCR as well as adverse pathological features, which might limit the statistical power of the study.

## Conclusion

In conclusion, hyperglycemia and hypertriglyceridemia were the two effective components of MetS that were identified as independent prognostic factors for BCRFS. The independently reduced association of hypertriglyceridemia with NOCD and ECE appear to a survival benefit, while the independently increased risk of upgrading points to a worse prognosis. Hyperglycemia and hypertriglyceridemia may become reliable and reversible predictors in predicting BCR and adverse pathological features of patients following RP that are benefit for helping clinicians improve patient counseling as well as risk-adapted strategies.

## Supplementary Information

**Additional file 1:**

## Data Availability

The raw data that support the findings of this study are available from the corresponding author upon reasonable request.

## Abbreviations

AJCC: American Joint Committee on Cancer
BCR: Biochemical recurrence
BCRFS: BCR-free survival
BMI: Body mass index
CI: Confidence interval
ECE: Extra-capsular extension
GS: Gleason score
HDL-C: High density lipoprotein cholesterol
HR: Hazard ratio
IQR: Interquartile range
ISUP: International Society of Urological Pathology
MetS: Metabolic syndrome
NOCD: Non-organ confined disease
OCD: Organ confined disease
OR: Odds ratio
PCa: Prostate cancer
PPC: Percentage of positive biopsy cores
PSA: Prostate specific antigen
PSM: Propensity score matching
PUTH: Peking University Third Hospital
Ref: Reference; RP: radical prostatectomy
RT: Radiation therapy
SVI: Seminal vesicle invasion
TG: Triglyceride
