# Supplementary material for: Metabolic syndrome and its components predict the biochemical recurrence and adverse pathological features of patients following radical prostatectomy: a propensity score matching study
Source: BMC Cancer. 2023 Jan 14;23:50. doi: 10.1186/s12885-023-10507-z (PMC9840841; doi:10.1186/s12885-023-10507-z)
Supplement: Supplementary file 1 — Additional file 1: [file 12885_2023_10507_MOESM1_ESM.docx]

**Supplementary Table 1** Subgroup analysis of effect of MetS and its components on BCRFS using the multivariate Cox proportional hazards model in RP patients after PSM.

| **Subgroups** | **MetS** | | **BMI** | | **Hypertension** | | **Hyperglycemia** | | **Hypertriglyceridemia** | | **Low HDL-C** | |
| --- | --- | --- | --- | --- | --- | --- | --- | --- | --- | --- | --- | --- |
|  | **HR (95%CI)** | **P value** | **HR (95%CI)** | **P value** | **HR (95%CI)** | **P value** | **HR (95%CI)** | **P value** | **HR (95%CI)** | **P value** | **HR (95%CI)** | **P value** |
| Age |  |  |  |  |  |  |  |  |  |  |  |  |
| <70 | 0.450 (0.092-2.186) | 0.322 | 1.686 (0.615-4.621) | 0.310 | 1.619 (0.486-5.398) | 0.433 | 2.590 (0.824-8.143) | 0.103 | 2.231 (0.662-7.515) | 0.195 | 0.519 (0.176-1.528) | 0.234 |
| ≥70 | 0.378 (0.093-1.539) | 0.175 | 3.128 (1.134-8.626) | 0.028 | 3.196 (0.873-11.706) | 0.079 | 1.597 (0.603-4.235) | 0.346 | 2.856 (1.002-8.142) | 0.050 | 1.373 (0.502-3.753) | 0.537 |
| BMI |  |  |  |  |  |  |  |  |  |  |  |  |
| BMI<25 | 0.337 (0.057-1.980) | 0.229 | - | - | 0.414 (0.098-1.746) | 0.230 | 3.421 (1.225-9.553) | 0.019 | 1.697 (0.489-5.889) | 0.405 | 1.017 (0.299-3.465) | 0.978 |
| BMI≥25 | 0.390 (0.111-1.372) | 0.143 | - | - | 2.053 (0.756-5.571) | 0.158 | 3.020 (1.264-7.215) | 0.013 | 4.093 (1.610-10.408) | 0.003 | 0.632 (0.254-1.574) | 0.324 |
| PSA |  |  |  |  |  |  |  |  |  |  |  |  |
| <20 | 0.300 (0.071-1.274) | 0.103 | 2.366 (0.870-6.437) | 0.092 | 1.968 (0.638-6.072) | 0.239 | 2.153 (0.826-5.611) | 0.117 | 3.703 (1.457-9.411) | 0.006 | 0.615 (0.221-1.709) | 0.351 |
| ≥20 | 0.473 (0.074-3.009) | 0.428 | 3.103 (0.928-10.374) | 0.066 | 1.476 (0.343-6.346) | 0.601 | 4.012 (1.102-14.607) | 0.035 | 1.747 (0.364-8.380) | 0.486 | 1.154 (0.343-3.882) | 0.817 |
| Pathologic T Stage |  |  |  |  |  |  |  |  |  |  |  |  |
| OCD | 0.357 (0.087-1.459) | 0.152 | 1.367 (0.478-3.908) | 0.559 | 2.320 (0.681-7.901) | 0.178 | 5.419 (1.763-16.653) | 0.003 | 3.586 (1.225-10.500) | 0.020 | 0.705 (0.274-1.812) | 0.468 |
| NOCD | 0.290 (0.063-1.338) | 0.113 | 3.243 (1.014-10.373) | 0.047 | 1.353 (0.401-4.560) | 0.626 | 1.559 (0.618-3.936) | 0.347 | 3.140 (0.910-10.834) | 0.070 | 0.703 (0.241-2.047) | 0.518 |
| Pathologic Gleason score | |  |  |  |  |  |  |  |  |  |  |  |
| Low (GS<8) | 0.459 (0.107-1.979) | 0.296 | 2.099 (0.691-6.381) | 0.191 | 2.544 (0.671-9.637) | 0.169 | 3.301 (1.018-10.700) | 0.047 | 4.777 (1.677-13.612) | 0.003 | 0.990 (0.378-2.597) | 0.984 |
| High (GS≥8) | 0.274 (0.061-1.225) | 0.090 | 2.177 (0.804-5.894) | 0.126 | 1.206 (0.411-3.545) | 0.733 | 1.880 (0.749-4.720) | 0.179 | 2.979 (0.862-10.296) | 0.085 | 0.529 (0.175-1.597) | 0.259 |
| Surgical margin |  |  |  |  |  |  |  |  |  |  |  |  |
| Negative | 0.292 (0.077-1.107) | 0.070 | 1.784 (0.679-4.685) | 0.240 | 1.859 (0.500-6.910) | 0.355 | 2.484 (0.928-6.650) | 0.070 | 4.232 (1.681-10.654) | 0.002 | 1.099 (0.436-2.773) | 0.841 |
| Positive | 0.875 (0.180-4.250) | 0.869 | 1.961 (0.600-6.410) | 0.265 | 1.278 (0.423-3.865) | 0.664 | 2.535 (0.994-6.464) | 0.051 | 1.788 (0.545-5.871) | 0.338 | 0.303 (0.081-1.143) | 0.078 |

BCRFS, BCR-free survival; BMI, body mass index; CI, confidence interval; GS, Gleason score; HDL-C, high density lipoprotein cholesterol; HR, hazard ratio; MetS, metabolic syndrome; NOCD, non-organ confined disease; OCD, organ confined disease; PSA, prostate specific antigen; PSM, propensity score matching; RP, radical prostatectomy.
